# *Rhizoma Dioscoreae* Extract Protects against Alveolar Bone Loss in Ovariectomized Rats via microRNAs Regulation

**DOI:** 10.3390/nu7021333

**Published:** 2015-02-16

**Authors:** Zhiguo Zhang, Changheng Song, Fangzhen Zhang, Lihua Xiang, Yanjing Chen, Yan Li, Jinghua Pan, Hong Liu, Gary Guishan Xiao, Dahong Ju

**Affiliations:** 1 Institute of Basic Theory, China Academy of Chinese Medical Sciences, Beijing 100700, China; E-Mails: zzgtcm@163.com (Z.Z.); sdsg_sch_86@126.com (C.S.); zfz13552961302@163.com (F.Z.); xlh891201@sina.com (L.X.); chenyj@163.com (Y.C.); lei_ruo@163.com (Y.L.); jh-p@163.com (J.P.); liuhong@163.com (H.L.); 2 School of Pharmaceutical Science, Dalian University of Technology, Dalian 116024, China; 3 Functional Genomics and Proteomics Laboratory, Osteoporosis Research Center, Creighton University Medical Center, Omaha, NE 68131, USA

**Keywords:** microRNA expression profile, herbal medicine, alveolar bone loss, ovariectomized rats, *Rhizoma Dioscoreae*

## Abstract

The aim of this study was to evaluate the osteoprotective effect of aqueous *Rhizoma Dioscoreae* extract (RDE) on the alveolar bone of rats with ovariectomy-induced bone loss. Female Wistar rats underwent either ovariectomy or sham operation (SHAM). The ovariectomized (OVX) rats were treated with vehicle (OVX), estradiol valerate (EV), or RDE. After treatments, the bone mineral density (BMD) and the three-dimensional microarchitecture of the alveolar bone were analyzed to assess bone mass. Microarrays were used to evaluate microRNA expression profiles in alveolar bone from RDE-treated and OVX rats. The differential expression of microRNAs was validated using real-time quantitative RT-PCR (qRT-PCR), and the target genes of validated microRNAs were predicted and further analyzed using Ingenuity Pathway Analysis (IPA). The key findings were verified using qRT-PCR. Our results show that RDE inhibits alveolar bone loss in OVX rats. Compared to the OVX rats, the RDE-treated rats showed upregulated expression levels of 8 microRNAs and downregulated expression levels of 8 microRNAs in the alveolar bone in the microarray analysis. qRT-PCR helped validate 13 of 16 differentially expressed microRNAs, and 114 putative target genes of the validated microRNAs were retrieved. The IPA showed that these putative target genes had the potential to code for proteins that were involved in the transforming growth factor (TGF)-β/bone morphogenetic proteins (BMPs)/Smad signaling pathway (*Tgfbr2/Bmpr2*, *Smad3/4/5*, and *Bcl-2*) and interleukin (IL)-6/oncostatin M (OSM)/Jak1/STAT3 signaling pathway (*Jak1*, *STAT3*, and *Il6r*). These experiments revealed that RDE could inhibit ovariectomy-induced alveolar bone loss in rats. The mechanism of this anti-osteopenic effect in alveolar bone may involve the simultaneous inhibition of bone formation and bone resorption, which is associated with modulation of the TGF-β/BMPs/Smad and the IL-6/OSM/Jak1/STAT3 signaling pathways via microRNA regulation.

## 1. Introduction

The quantity and quality of alveolar bone has long been the focus of odontologists and implantologists [1]. Alveolar bone is essential for the support of teeth, which are anchored to the bone by desmodontal fibers. Previous research has shown that postmenopausal women suffering from systemic bone loss and osteoporosis takes a high risk of alveolar bone loss which is associated with tooth loss and periodontal disease [2,3]. This alveolar bone loss characterized by reduced alveolar bone mass and altered alveolar bone structure [4] occurs rapidly during the early postmenopausal period, and levels off approximately six years after menopause, likely due to the decrease in estrogen production in postmenopausal women [5].

Among many mechanisms of metabolic bone diseases, microRNAs (miRNAs) regulation plays a unique role in bone remodeling [6,7]. More and more miRNAs associated with regulation of bone metabolism are found. miRNAs are small non-coding RNAs characterized by a length of 18–25 nucleotides and capable of binding to complementary 3′UTR regions of their target genes, thereby modulating the transcription of the target mRNA [8]. miRNAs have been implicated in a number of basic metabolic pathways and biological processes [9], including osteoporosis pathways [10]. For instance, miR-138 displays inhibitory action on bone formation *in vivo* by attenuating the focal adhesion kinase signaling pathway [11]. Furthermore, several lines of evidence show that the modulation of miRNAs could provide therapeutic benefits against bone loss [12]. For example, miR-21 may contribute to bone formation by regulating TNF-α in estrogen deficiency-induced osteoporosis [13].

*Rhizoma Dioscoreae* (RD) is the dried rhizoma of *Dioscorea opposita* Thunb, which is widely distributed and cultivated in East Asia, including China, Korea, and Japan, *etc.* [14]. RD has long been used in foods and oriental folk medicine. Many studies reported that RD has various beneficial activities and nutritional superiority [15,16]. Meanwhile, our previous study indicated that treatment with *Rhizoma Dioscoreae* extract (RDE) protects against alveolar bone loss of ovariectomized (OVX) rats via regulating *Wnt* and p38 MAPK signaling [17]. In light of the important role of miRNAs in bone remodeling, we question whether the protective effect of RDE on alveolar bone loss is associated with miRNAs. To figure out this question, this study was performed to evaluate modulatory effect of RDE on miRNAs expression in alveolar bone.

## 2. Experimental Section

### 2.1. Preparation of Aqueous Extract

Preparation and high-performance liquid chromatography analyses of RDE were performed as we previously reported [18]. In this study, we used the same batch of RDE as presented previously [17], but the results of this study were not from the previously reported work.

### 2.2. Animal Grouping and Treatments

Many studies have used 6-month-old female rats that have undergone a bilateral ovariectomy to model postmenopausal osteoporosis [19,20]. We obtained a total of forty-eight 6-month-old female Wistar rats with an average body weight of 300 ± 20.0 g from the Experimental Animal Center of the Academy of Military Medical Sciences (SCXK-(Military) 2013-004, Beijing, China). This experimental research on animals was approved by The Institutional Ethics Committee of institute of basic theory, China Academy of Chinese Medical Sciences (Approval number: 2013-009). The acclimatized rats were either bilaterally OVX (*n* = 36) or Sham-operated (SHAM, *n* = 12) [21]. The OVX rats were randomly divided into three groups: OVX group (OVX, *n* = 12); estradiol valerate treatment group (EV, *n* = 12); RDE group (RDE, *n* = 12). Estradiol valerate (1 mg/tablet, Bayer China Ltd., Shanghai, China) was dissolved in distilled water. The rats in the EV group were treated with estradiol valerate (0.1 mg/kg body weight) daily by oral gavage. The rats in the RDE group were treated with RDE dissolved in distilled water at 1.3 g/kg body weight/day by oral gavage. The gavage dosage was based on the recommended dosage for humans (30 g/day) according to Chinese Pharmacopeia, adjusted for the rat/human body mass ratio. The rats in the OVX and the SHAM groups were administered the same volume of distilled water by oral gavage. All rats were fed standard chow during the course of the experiments (Animal Center of the Fourth Military Medical University, Xi’an, China). All treatments started 1 week after OVX surgery, and lasted for 12 weeks. No animal died in 12-week treatment period.

### 2.3. Preparation of Specimens

The day after the last treatment, the animals were anesthetized using ketamine (80 mg/kg body weight) and xylazine (12 mg/kg body weight) by intraperitoneal injection and sacrificed by exsanguination. The right mandibles were dissected and stored at −20 °C for measurements of bone mineral density (BMD) and microstructure by micro-computerized tomography (micro-CT). The left mandibles were dissected, so only the bone tissue between molars and incisor remained, and then stored at −80 °C for microarray and real-time quantitative RT-PCR (qRT-PCR) assays.

### 2.4. Micro-CT Analysis

Without sample preparation, the right mandible of each animal was scanned using a high-resolution micro-CT (Skyscan 1172 micro-CT system, Antwerp, Belgium). The micro-CT determines the conical geometry of the x-ray source by a cone-beam reconstruction method. The Skyscan 1172 micro-CT system was used according to a method previously described [22]. Each sample was scanned with a desired resolution (6.8 μm). A low-pass filter was used to remove noise from the resulting gray-scale images. The trabecular bone was measured using a fixed threshold.

The images of mandible were captured at a voltage of 100 keV and 100 μA current. A cubic region (1.0 mm × 1.0 mm × 1.0 mm) beginning 1.5 mm beneath the lowest point of first molar crown was reconstructed as the “volume of interest” (VOI). The standard Skyscan software package was used to performed morphological measurements of the trabecular bone within the VOI. We used three-dimensional analyses to assess the BMD, the trabecular bone volume fraction (BV/TV), the trabecular number (Tb.N), the trabecular thickness (Tb.Th), the trabecular separation (Tb.Sp), the structural model index (SMI), and the degree of anisotropy (DA) for the same VOI [23].

### 2.5. miRNA Microarray Data Analysis

Alveolar bone was prepared from six rats of RDE group and six rats of OVX group. The miRNA microarray assay was conducted by KangChen Bio-tech (Shanghai, China). TRIzol (Invitrogen, Carlsbad, CA, USA) and the RNeasy Mini Kit (Qiagen, Valencia, CA, USA) were used to harvest total RNA, which subsequently were labeled with miRCURY Hy3/Hy5 Power labeling kit (Exiqon, Vedbaek, Denmark) and hybridized on the miRCURY LNA Array (v.11.0) (Exiqon, Vedbaek, Denmark). Axon GenePix 4000B microarray scanner (Axon Instruments, Foster City, CA, USA) was used to scan slides. The raw intensity of the image was read using GenePix Pro V6.0 (Molecular Devices, Sunnyvale, CA, USA). For obtaining a mean intensity, we calculated the intensity of green signal by background subtraction, and further averaged replicated spots. The data were normalized using the median normalization method: normalized data = (foreground − background)/median. Significance of the results was determined via fold change and *t*-test. We used threshold values (fold change ≥ 2 and *p* value < 0.05) to screen differentially expressed miRNAs.

### 2.6. RNA Isolation and qRT-PCR

qRT-PCR analysis for miRNAs was performed using an ABI 7500 system (Applied Biosystems, Foster City, CA, USA) and SYBR RT-PCR kits (Takara, Dalian, China). An internal control, U6, was used to normalized the relative expression level of miRNAs by the 2^−ΔΔCt^ cycle threshold method. In Table 1, the primers for detecting all the miRNAs are described. For target mRNA analysis, the primers are listed in Table 2. mRNA expression data were normalized by the level of U6 or *Gapdh* expression.

**Table 1:** Primers of miRNAs.

Name	Primers
U6	F: 5′-GCTTCGGCAGCACATATACTAAAAT-3′ R: 5′-CGCTTCACGAATTTGCGTGTCAT-3′
rno-miR-500-3p	GSP: 5′-GGAAGGCACCTGGGCAAG-3′ R: 5′-GTGCGTGTCGTGGAGTCG-3′
rno-miR-499-3p	GSP: 5′-GGGGAACATCACAGCAAGTC-3′ R: 5′-GTGCGTGTCGTGGAGTCG-3′
rno-miR-214-3p	GSP: 5′-GGGGACAGCAGGCACAGAC-3′ R: 5′-GTGCGTGTCGTGGAGTCG-3′
rno-miR-20b-5p	GSP: 5′-GGGGCAAAGTGCTCATAGTG-3′ R: 5′-GTGCGTGTCGTGGAGTCG-3′
rno-miR-877	GSP: 5′-GGGGAAGTAGAGGAGATGGC-3′ R: 5′-GTGCGTGTCGTGGAGTCG-3′
rno-miR-451-5p	GSP: 5′-GGGGGAAACCGTTACCATTAC-3′ R: 5′-GTGCGTGTCGTGGAGTCG-3′
rno-miR-3577	GSP: 5′-GGGTTCTGTCCCTCTTGGC-3′ R: 5′-GTGCGTGTCGTGGAGTCG-3′
rno-miR-370-3p	GSP: 5′-AGCCTGCTGGGGTGGAA-3′ R: 5′-GTGCGTGTCGTGGAGTCG-3′
rno-miR-181d-5p	GSP: 5′-GGGGCATTCATTGTTGTCG-3′ R: 5′-GTGCGTGTCGTGGAGTCG-3′
rno-miR-23b-3p	GSP: 5′-GGGATCACATTGCCAGGG-3′ R: 5′-GTGCGTGTCGTGGAGTCG-3′
rno-miR-191a-5p	GSP: 5′-GGCAACGGAATCCCAAAAG-3′ R: 5′-GTGCGTGTCGTGGAGTCG-3′
rno-miR-200c-3p	GSP: 5′-GGGGTAATACTGCCGGGTAA-3′ R: 5′-GTGCGTGTCGTGGAGTCG-3′
rno-miR-328a-3p	GSP: 5′-AACTCGCCCTCTCTGCCC-3′ R: 5′-GTGCGTGTCGTGGAGTCG-3′

**Table 2:** Primers of mRNA targets.

Name	Primers
*Gapdh*	F: 5′-GGAAAGCTGTGGCGTGAT-3′ R: 5′-AAGGTGGAAGAATGGGAGTT-3′
*Bmpr2*	F: 5′-CAACACCACTCAGTCCGCC-3′ R: 5′-GCTCCAGCAGCTTCAGGTTAT-3′
*Jak1*	F: 5′-TGATGAATAACGACCACCAAAA-3′ R: 5′-TCCTACTAGGGAGCAGGGATAG-3′
*STAT3*	F: 5′-GAAAAGGACATCAGTGGCAAGA-3′ R: 5′-GGAATGTCAGGGTAGAGGTAGACC-3′
*Tgfbr2*	F: 5′-TGTGGAGGAAGAACGACAAGAA-3′ R: 5′-AGAGTGAAGCCGTGGTAGGTG-3′
*Il6*	F: 5′-TGCCTTCTTGGGACTGATGT-3′ R: 5′-ATACTGGTCTGTTGTGGGTGGT-3′
*Smad3*	F: 5′-CTGGCTCCGGTAAAGGATTG-3′ R: 5′-ATGGGCTCCTCATTTCACAAC-3′
*Smad4*	F: 5′-TGCCTCAGTGACCACGCC-3′ R: 5′-CCCAGGACCAGGGATGTTTC-3′
*Smad5*	F: 5′-ACAGACCCTGCCAATAACAAGA-3′ R: 5′-CACTAAGACACTCGGCATACACC-3′
*Il6r*	F: 5′-CTATGGCAACCTTAGTGCTCATT-3′ R: 5′-TGAGGTATTCTGCTTAACGGATG-3′
*Kras*	F: 5′-CAGTAGACACGAAACAGGCTCA-3′ R: 5′-CTTTTTCCCATCTTTGCTCATC-3′
*Bcl2*	F: 5′-TGGGATGCCTTTGTGGAAC-3′ R: 5′-CATATTTGTTTGGGGCAGGTC-3′

### 2.7. Western Blotting

Alveolar bone were crushed in liquid nitrogen to extract bone proteins, which were further solubilized by radioimmunoprecipitation assay (RIPA) buffer containing protease inhibitors and phosphatase inhibitors. Insoluble substance was separated and removed by centrifugation at 10,000 rpm for 5 min at 4 °C. BCA reagent (Pierce, Rockford, IL, USA) was used to determine protein concentrations. Dissolved 80 μg proteins extracted from each sample were used for electrophoresis in 15% SDS-polyacrylamide gels, and transferred onto polyvinylidene fluoride membranes (Immobilon-P, Millipore, Bedford, MA, USA). After blocked with 5% nonfat dry milk in TBST for 1 h, the membranes were further incubated with antibodies against Smad4 (1:1000 dilution, Santa Cruz Biotechnology, Santa Cruz, CA, USA), STAT3 (1:1000 dilution, Santa Cruz Biotechnology) and beta-actin (1:25,000 dilution, Sigma-Aldrich, St. Louis, MO, USA) overnight at 4 °C. Subsequently, membranes were washed with TBST and incubated with a HRP-linked antibody (1:1000 dilution, Cell Signaling Technology, Beverly, MA, USA) for 1 h. For detection of Immunoreactive proteins, an enhanced chemiluminescence kit (PerkinElmer, Waltham, MA, USA) was used. Using Quantity One software (Bio-Rad, Hercules, CA, USA), the intensities of specific bands were quantified with densitometry. Quantified intensities were further normalized for beta-actin and expressed as fold increase *versus* Sham or OVX control.

### 2.8. Ingenuity Pathway Analysis (IPA)

To analyze the miRNA microarray data and reveal the underlying biological processes of specific gene, IPA (Ingenuity^®^ Systems, Redwood, CA, USA) was used for the identification of the potential mRNA targets, function and pathway analysis.

Differentially expressed miRNAs between the RDE group and the OVX group were imported into IPA. We set confidence to “highly predicted” and “experimental observed” and predicted putative miRNA targets in rats using an integrated database including TargetScan, TarBase, miRecords and the Ingenuity^®^ Knowledge Base. Furthermore, identified mRNA targets underwent a canonical pathway analysis, and the top canonical pathways associated with our data were retrieved using the core-analysis in IPA.

### 2.9. Statistical Analysis

All values were expressed as the mean ± standard deviation. All analyses were conducted using the SPSS 13.0 (SPSS Inc., Chicago, IL, USA). The difference of the evaluated parameters between the groups was tested using the analysis of variance (ANOVA) followed by the least significant difference (LSD) test. The data from all groups passed the normality test by Kolmogorov–Smirnov statistics. *P* < 0.05 was considered to be statistically significant.

## 3. Results

### 3.1. Effect of RDE on Bone Mineral Density and Trabecular Bone Microarchitecture

**Figure 1:**
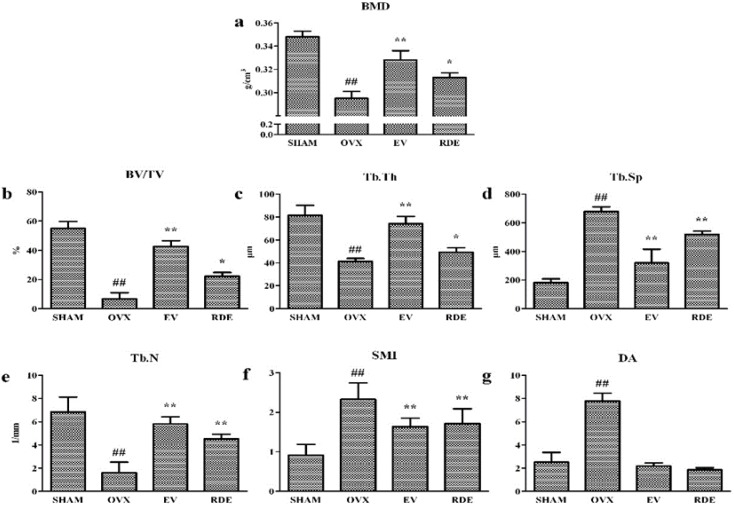
Effect of *Rhizoma Dioscoreae* extract (RDE) on the bone mineral density (BMD) and trabecular bone microarchitecture after 12 weeks treatment. (**a**) BMD, (**b**) trabecular bone volume fraction (BV/TV), (**c**) trabecular thickness (Tb.Th), (**d**) trabecular separation, (**e**) trabecular number (Tb.N), (**f**) structural model index (SMI), (**g**) degree of anisotropy (DA). SHAM: Sham-operated group; OVX: ovariectomized group; EV: estradiol valerate treatment group; RDE: RDE treatment group. ^#^ *P* < 0.05; ^##^ *P* < 0.01, *vs.* SHAM group; * *P* < 0.05; ** *P* < 0.01, *vs.* OVX group.

**Figure 2:**
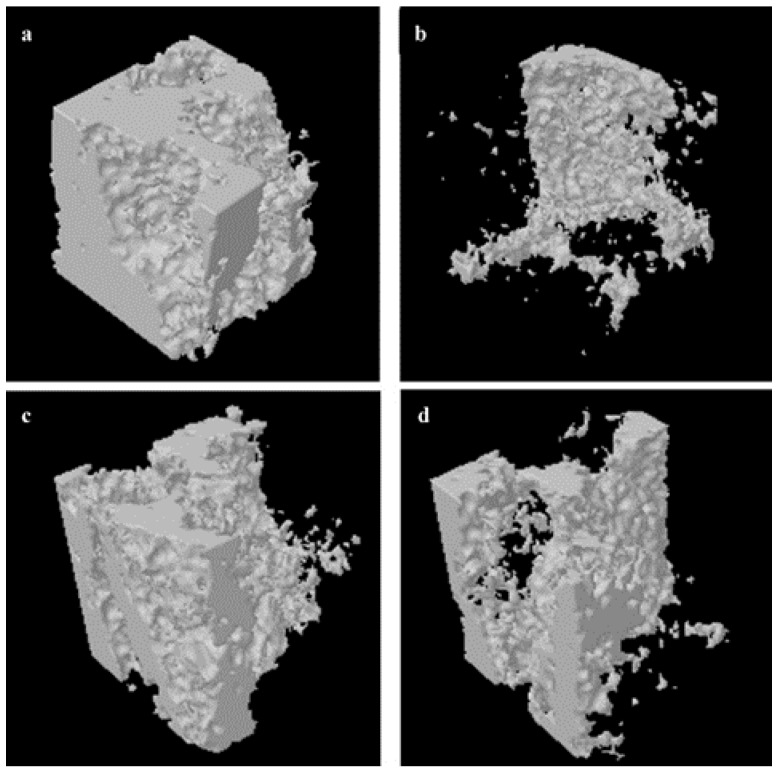
Representative sample from each group: 3-D architecture of alveolar bone beneath the lowest point of first molar crown. (**a**) Sham-operated group (SHAM), (**b**) ovariectomized group (OVX), (**c**) estradiol valerate treatment group (EV), (**d**) *Rhizoma Dioscoreae* extract treatment group (RDE).

We evaluated the trabecular bone microarchitecture of six groups using micro-CT. Analysis of the morphometric parameters from alveolar bone indicated that ovariectomy significantly decreased the BMD, BV/TV, Tb.N, and Tb.Th (*P* < 0.01), and increased Tb.Sp, SMI, and DA (*P* < 0.01) compared to the SHAM group. Treatment with RDE or EV significantly inhibited the OVX-induced changes (Figure 1a–g). Further, treatment with EV or RDE relieved damage to the trabecula in alveolar bone induced by the ovariectomy (Figure 2a–d).

### 3.2. Effect of RDE on miRNAs Expression Profile

The array results demonstrated that the expression of 16 miRNAs was altered (≥2-fold) between the alveolar bone from RDE and OVX group rats. Specifically, 8 miRNAs were upregulated and 8 miRNAs were downregulated (Table 3).

**Table 3:** Differentially expressed microRNAs between the *Rhizoma Dioscoreae* extract treatment group (RDE) and ovariectomized group (OVX).

microRNA	Fold Change	*P* Value
	*Upregulated miRNAs*	
rno-miR-500-3p	15.891	0.038
rno-miR-499-3p	11.323	0.043
rno-miR-382-3p	8.130	0.000
rno-miR-214-3p	3.256	0.043
rno-miR-20b-5p	3.026	0.011
rno-miR-23b-3p	2.633	0.044
rno-miR-877	2.574	0.044
rno-miR-423-5p	2.548	0.023
	*Downregulated miRNAs*	
rno-miR-191a-5p	−2.266	0.033
rno-miR-181d-5p	−2.370	0.012
rno-miR-200c-3p	−3.498	0.009
rno-miR-328a-3p	−4.116	0.046
rno-miR-451-5p	−4.423	0.029
rno-miR-124-5p	−4.908	0.010
rno-miR-3577	−6.823	0.035
rno-miR-370-3p	−12.780	0.007

### 3.3. Confirmation of Differential Levels of miRNA Expression by qRT-PCR

**Figure 3:**
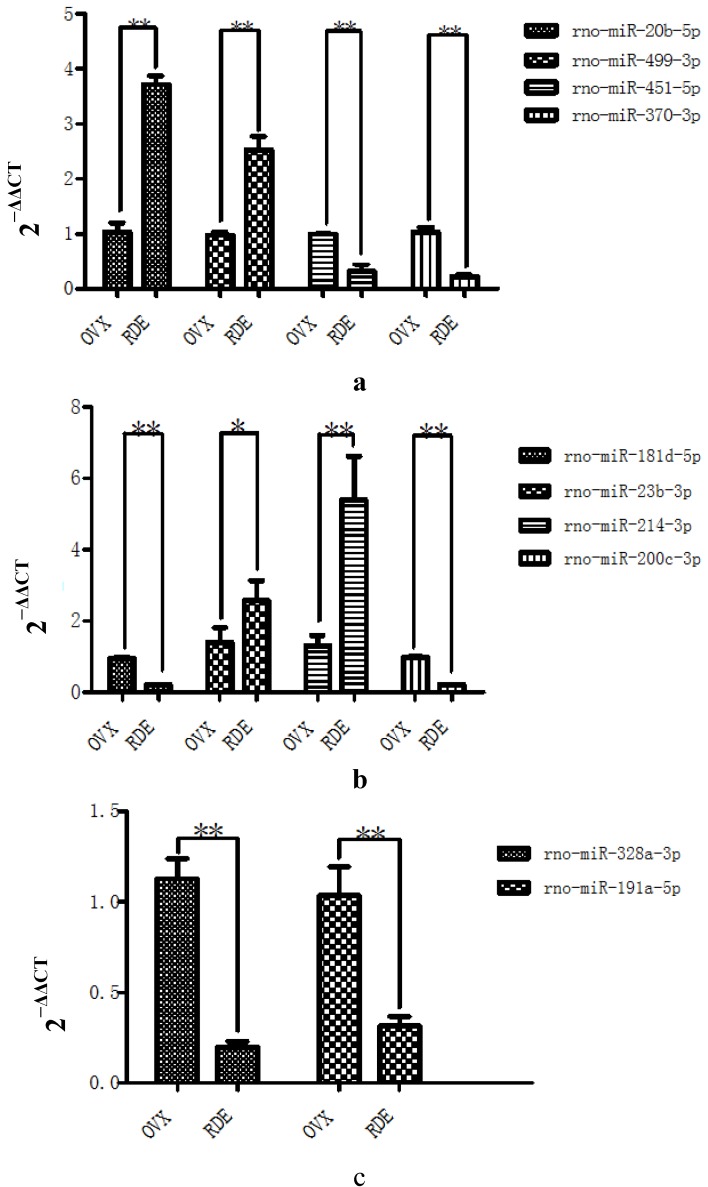

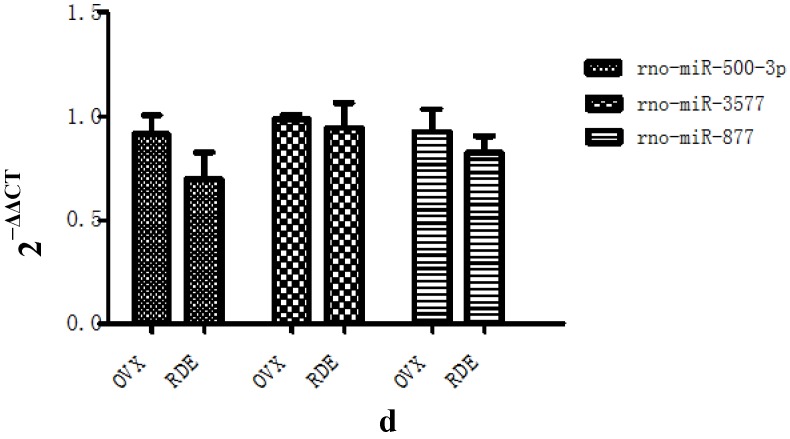
Validation of the differential expression of 13 miRNAs identified in the microarray by qRT-PCR: (**a**)–(**d**) effect of RDE on the expression of 13 miRNAs (*n* = 6 in each group), respectively. * *P* < 0.05; ** *P* < 0.01, *vs.* OVX group.

qRT-PCR analysis was used to verify the differential expression of 13 of the 16 identified miRNAs. The comparative changes in these miRNAs, as determined by microarray and qRT-PCR, are presented in Figure 3a–d. In nearly all cases, the results of the qRT-PCR analysis generally agreed with the changes in the microarray analysis, with the exception of rno-miR-500-3p, rno-miR-877, and rno-miR-3577. There was no significant difference in the expression of rno-miR-500-3p, rno-miR-877, and rno-miR-3577 between RDE group and OVX group.

### 3.4. Putative miRNA Targets

Validated differentially expressed miRNAs were imported into IPA. One hundred fourteen putative target genes of differentially expressed miRNAs are listed in the supplementary materials.

### 3.5. Pathway Analysis of Putative Target Genes

We characterized putative target genes by signaling pathways and function using the IPA software (Ingenuity Systems, Redwood City, CA, USA) from Ingenuity Pathways Knowledge Base (IPKB) (http://www.ingenuity.com). IPA could rank signaling pathways according to scores, which are calculated based on the significance of the involved genes. Using IPKB, the differential expression genes were allocated into pathways. Of these ranked pathways, three pathways were identified with significance values of less than 1.5 × 10^−4^ (Table 4).

**Table 4:** Canonical pathways associated with target genes.

Ingenuity Canonical Pathways	*P* Value
TGF-β signaling	9.02 × 10^−6^
Oncostatin M signaling	2.30 × 10^−5^
Role of JAK family kinases in IL-6-type cytokine signaling	1.30 × 10^−4^

### 3.6. Confirmation of Differential Levels of Target Genes Expression by qRT-PCR

**Figure 4:**
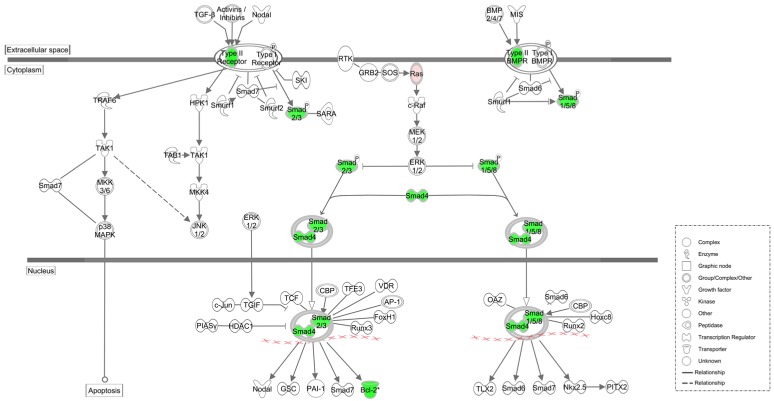
Schematic diagram of TGF-β and BMPs signaling pathways in the anti-osteopenic effect of RDE: Down-regulated genes are in green, and up-regulated genes are in red. Genes in white were not specified but were used to establish network based on relationships.

**Figure 5:**
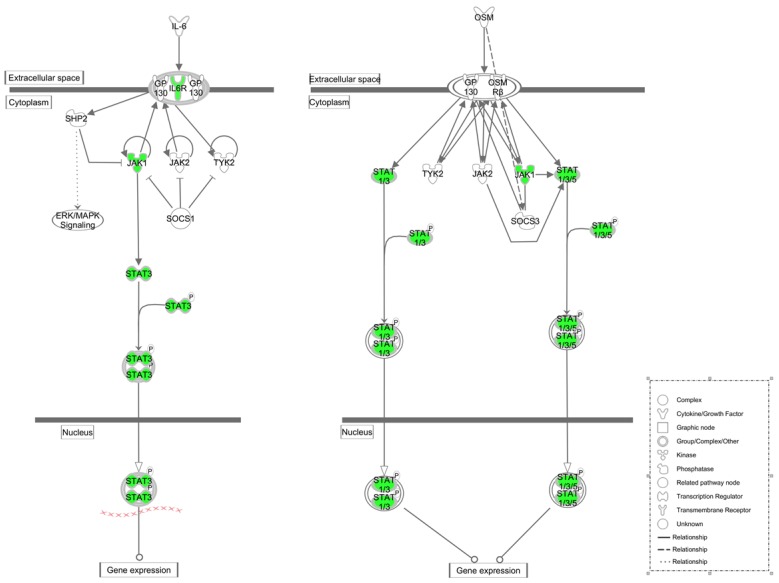
Schematic diagram of IL-6 and OSM signaling pathways in the anti-osteopenic effects of RDE: Down-regulated genes are in green. Genes in white were not specified but were used to establish network based on relationships.

**Figure 6:**
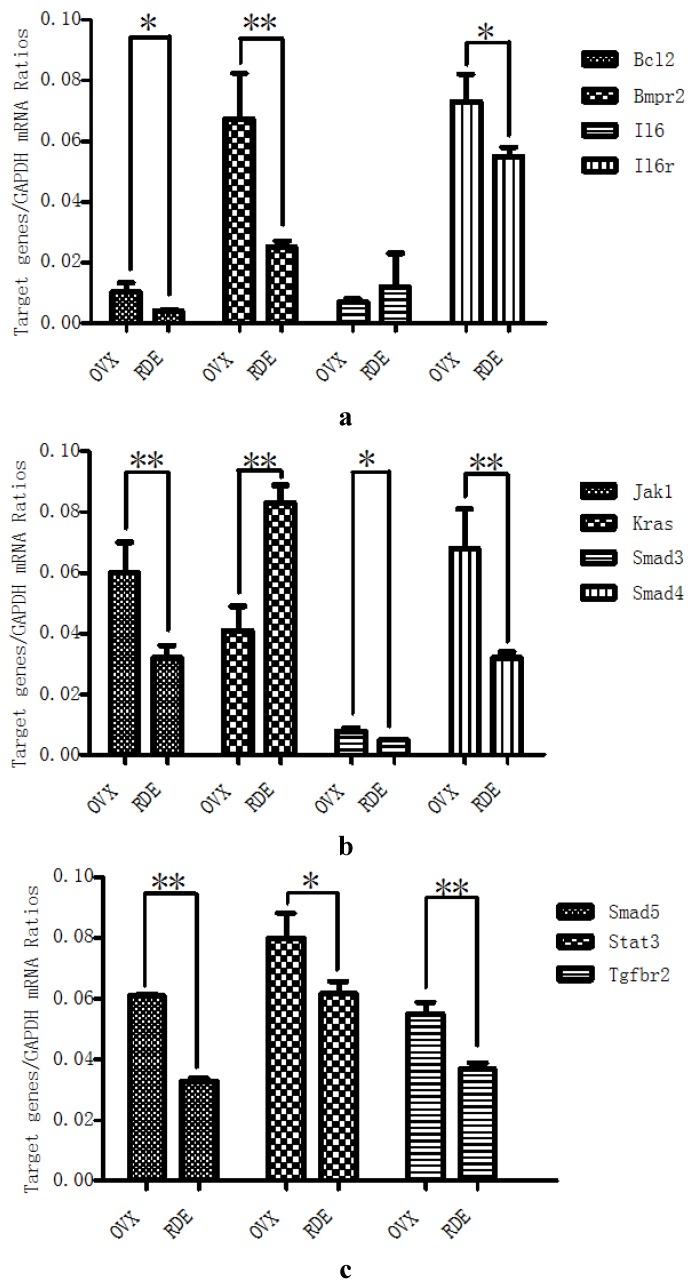
Validation of 11 target genes by qRT-PCR: Effect of RDE on the expressions of *Bmpr2*, *Il6, Il6r*, and *Bcl2* (**a**); *Jak1*, *Kras*, *Smad3*, and *Smad4* (**b**); and *Smad5*, *STAT3*, and *Tgfbr2* (**c**). * *P* < 0.05; ** *P* < 0.01, *vs*. OVX group.

We next assessed changes in the mRNA expression of 11 genes involved in the pathways identified by IPA (Figure 4 and Figure 5). These genes were as follows: bone morphogenetic protein receptor, type II (*Bmpr2*), Janus kinase 1 (*Jak1*), signal transducer and activator of transcription 3 (*STAT3*), transforming growth factor, beta receptor II (*Tgfbr2*), Kirsten rat sarcoma viral oncogene (*Kras*), interleukin 6 (*Il6*), SMAD family member 3 (*Smad3*), SMAD family member 4 (*Smad4*), SMAD family member 5 (*Smad5*), interleukin 6 receptor (*Il6r*), and B-cell CLL/lymphoma 2 (*Bcl2*). We found that changes in gene expression agreed with the predictions except Il6, and were downregulated or upragulated in the RDE treated rats (Figure 6a–c).

### 3.7. Confirmation of Proteins by Western Blotting

**Figure 7:**
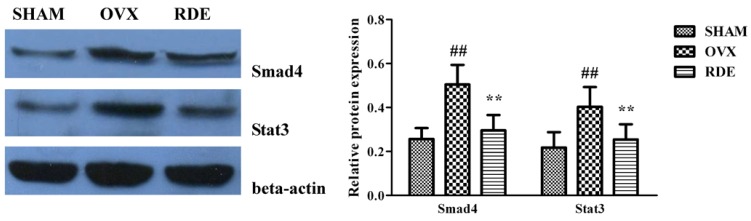
Validation of two proteins by western blotting: Effect of RDE on the expressions of Smad4 and STAT3. Beta-actin was used as the reference gene. Representative Western blot bands are shown in left panel. Band intensities were quantified using densitometry (right panel). ^#^ *P* < 0.05; ^##^ *P* < 0.01, *vs.* SHAM group; * *P* < 0.05; ** *P* < 0.01, *vs.* OVX group.

Changes in protein expression of *Smad4* and *STAT3* were further assessed. We found that changes in protein expression of two genes agreed with the mRNA expression, and were downregulated in the RDE treated rats (Figure 7).

## 4. Discussion

After ovariectomy, BMD of alveolar bone in rats was markedly reduced, which was caused by an increase in alveolar bone turnover in the OVX rats compared to the Sham rats. In contrast, treatment with the RDE increased the BMD of the alveolar bone compared to the OVX group. The analysis of 3-D bone microarchitecture by micro-CT showed that the alveolar bone in RDE or EV-treated rats had less bone loss than the OVX group. The significant change in BV/TV, Tb.Th, Tb.N, Tb.Sp and SMI reflected this anti-osteopenic effect of RDE and EV, but EV had a more potent effect than RDE.

To explore the mechanism of the anti-osteopenic effect of RDE on alveolar bone, we screened the differential expression of miRNAs by microarray, identified putative miRNA target genes, and found three key pathways with IPA. The miRNA microarray and IPA method may provide a powerful tool for discovery of new clinical treatment. In our study, we succeeded in identifying 16 miRNAs, which were differentially expressed in the alveolar bone of the RDE group compared to the OVX group, and 13 of these miRNAs were validated by qRT-PCR. Based on the 13 validated miRNAs, 114 putative target genes were retrieved from an integrated database with IPA. Table 2 shows the top three canonical pathways associated with putative target genes.

The transforming growth factor-beta (TGF-β) superfamily had more than forty members, including TGF-βs, Nodal, activin, and bone morphogenetic proteins (BMPs) [24]. Canonical TGF-β/BMPs/Smads pathway transmit signals across the plasma membrane by the formation of heteromeric complexes of specific type I (*Tgfbr1/Bmpr1*) and type II (*Tgfbr2/Bmpr2*) serine/threonine kinase receptors. *Tgfbr1/Bmpr1* is phosphorylated following the activation of specific *Tgfbr2/Bmpr2* [25]. Activated *Tgfbr1/Bmpr1* then triggers intracellular signaling through phosphorylating receptor-activated Smads, (R-Smads, Smad1-3, 5, 8). Activated R-Smads and common-mediator Smads (Co-Smads, Smad4) form a complex and then translocate into the nucleus to initiate transcription [26].

TGF-β1 is one of the most abundant growth factors in human bone and is expressed through all stages of alveolar bone formation [27]. Despite conflicting results, most data supports a model wherein TGF-β1 enhances bone formation by promoting proliferation and differentiation of mesenchymal cells, recruitment of preosteoblast, differentiation of osteoblast (but inhibition of terminal differentiation), and production of bone matrix [28,29]. The Bcl-2 family was clearly associated with apoptosis inhibition [30]. TGF-β1 could suppress osteoblast or osteoclast apoptosis by increasing expression of Bcl-2 family members [31,32].

BMPs exhibit versatile functions in mammalian development. The roles of BMPs in bone formation are widely recognized. BMPs increase bone formation mainly by promoting migration, proliferation, and differentiation of osteoprogenitors and matrix synthesis [33,34].

RDE increased the expression of rno-miR-20b-5p and rno-miR-23b-3p in alveolar bone after 12 weeks of treatment (Figure 3a–d), which could result in the down-regulation of target gene expression, including *Tgfbr2/Bmpr2*, *Smad3/4/5*, *Bcl2*. Our qRT-PCR analyses of *Tgfbr2/Bmpr2*, *Smad3/4/5*, *Bcl2* expression (Figure 6a–c) and western analysis of SMAD4 (Figure 7) validated this prediction. The down-regulation of *Tgfbr2/Bmpr2*, *Smad3/4/5* could inhibit TGF-β/BMPs/Smad signaling, leading to a reduction in osteoblastogenesis. Alternatively, down-regulation of *Bcl2* could promote osteoblast or osteoclast apoptosis, which might cause a dual inhibition of RDE on bone formation and resorption in the alveolar bone of OVX rats.

IL-6 family of cytokines include IL-6, IL-11, leukemia inhibitory factor (LIF), oncostatin M (OSM), ciliaryneurotrophic factor (CNTF), and cardiotrophin-1 (CT-1) [35]. The receptors of the IL-6 family cytokines have a specific ligand-binding subunit and a common signal-transducing subunit, GP130. IL-6 family cytokines binding to their receptors trigger the dimerization of GP130. Subsequently, these homodimers activate the associated Janus kinases (JAK1, JAK2, and TYK2) [36], which promote further activation of the members of signal transducer and activator of transcription (STATS) family [37].

IL-6 shows dual effect on bone metabolism. On the one hand, IL-6 has a stimulatory action on mesenchymal progenitor differentiation to the osteoblastic lineage [38] and potently inhibits the apoptosis of osteoblasts. On the other hand, IL-6 can stimulate osteoclastogenesis and bone resorption [39] by facilitating interactions between osteoblasts and osteoclasts [40], which is the main effect of IL-6 on bone.

OSM transduces signaling via a receptor complex containing either the LIF receptor (LIFR) or OSM receptor (OSMR). OSM specifically mediated sclerostin expression and bone formation by the LIFR [41], while it mediated osteoblast/adipocyte commitment by the OSMR [42]. OSM shows a unique receptor-specific divergent effect on osteoblasts and osteoclasts [43].

A 12-week treatment with RDE increased the expression of rno-miR-20b-5p and rno-miR-23b-3p and decreased the expression of rno-miR-191a-5p in alveolar bone (Figure 3a–d), which could result in the down-regulation of *Jak1*, *STAT3*, *Il6r* and up-regulation of *Il6*. With the exception of *Il6*, the change in gene expression was validated by qRT-PCR analyses (Figure 6a–c). The change in protein expression of *STAT3* was validated by western analysis (Figure 7). We found that RDE inhibits IL-6/OSM signaling by regulating miRNA expression and attenuating the expression of *Jak1* and *STAT3*, two key kinases of intracellular signal transduction. This effect may cause a synchronous inhibition of bone formation and resorption in alveolar bone of OVX rats.

In summary, we observed that the expression of *Tgfbr2/Bmpr2*, *Smad3/4/5*, *Bcl-2*, *Jak1*, *STAT3*, and *Il6r* in the alveolar bone was downregulated after treatment with RDE (Figure 4 and Figure 5) and these genes were associated with the TGF-β/BMPs/Smad signaling and IL-6/OSM/Jak1/STAT3 signaling. This inhibitory effect of RDE occurred via the regulation of miRNA expression (rno-miR-20b-5p, rno-miR-23b-3p and rno-miR-191a-5p) and lead to a down-regulation of high rate of bone turnover resulted from ovariectomy. Combined with results of present and previous studies [17,18], we found RDE had protective effect on systemic and alveolar bone loss, but the mechanisms were not the same. In alveolar bone, RDE inhibited the alveolar osteopenia not only via regulating Wnt and p38 MAPK signaling but also via regulating expression of several miRNAs.

In this research, we only explored the anti-osteopenic effect of RDE in an animal model. A line of studies had to be performed before RDE became a promising alternative therapeutic agent or functional food for relieving alveolar bone loss of postmenopausal women.

## 5. Conclusions

RDE may inhibit ovariectomy-induced alveolar bone loss in rats. The mechanism for the anti-osteopenic effect of RDE may lie in the simultaneous inhibition of both bone formation and bone resorption that occurs following regulation of miRNA expression, which modulated TGF-β/BMPs/Smad signaling and IL-6/OSM/Jak1/STAT3 signaling. Our study indicates that the aqueous extract of *Rhizoma Dioscoreae* may have potential use as a drug or functional food for alveolar bone loss in postmenopausal females.

## Supplementary Files

Supplementary File 1
